# Supplementary material for: Global Hemostasis Potential in COVID-19 Positive Patients Performed on St-Genesia Show Hypercoagulable State
Source: J Clin Med. 2022 Dec 7;11(24):7255. doi: 10.3390/jcm11247255 (PMC9785526; doi:10.3390/jcm11247255)

a) ROC of Inh. ETP (%) (Covid-19 + (severe & non-severe)) VS (Covid-19 -)

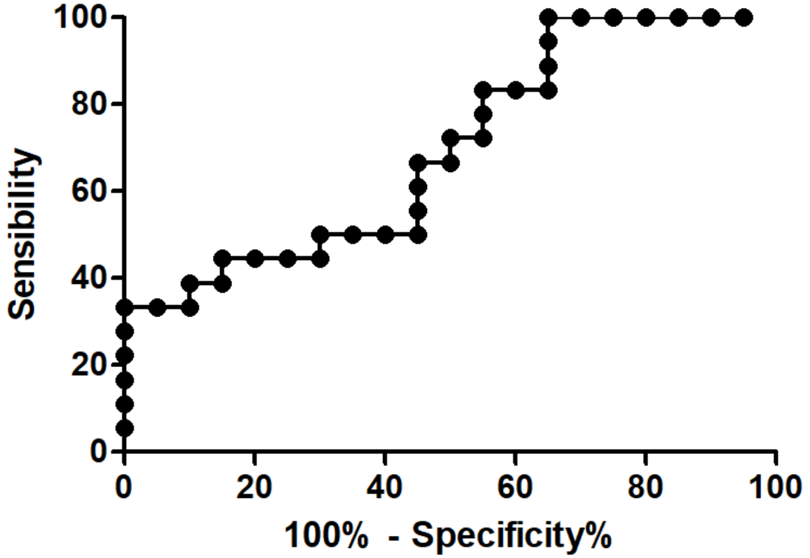

b) ROC curve of inh. ETP (%) (Covid-19 + non-severe and Covid-19 -) VS (Covid-19 + severe)

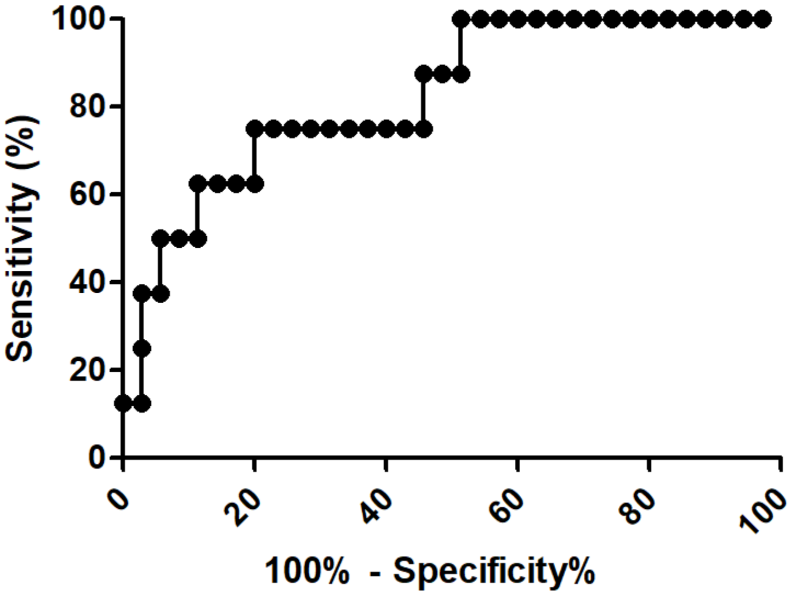

Supplement: Supplementary file 1 [file jcm-11-07255-s001.zip › jcm-2054612-supplementary-Figure S1.pdf]
